# COVID-19 Adenoviral Vaccine-Induced Immune Thrombotic Thrombocytopenia (VITT), COVID-19-Related Thrombosis, and the Thrombotic Thrombocytopenic Syndromes

**DOI:** 10.3390/hematolrep14040050

**Published:** 2022-12-01

**Authors:** Gewil Daniella Olipas Allas, Joekeem Del Rosario Arizala, Rafael Vincent Mercado Manalo

**Affiliations:** 1 Department of Biochemistry, The Graduate Center, The City University of New York (CUNY), New York, NY 10016, USA; 2 Department of Biochemistry and Molecular Biology, College of Medicine, University of the Philippines Manila, Ermita, Manila 1000, Philippines

**Keywords:** thrombocytopenia, VITT, HIT, TTP, COVID-19

## Abstract

Adenoviral-based vaccines such as ChadoX1 CoV-19 (AstraZeneca) and Ad26.COV2.S (J&J) were developed to prevent infection and reduce hospitalization or death in Coronavirus Disease 2019 (COVID-19) patients. Although these vaccines passed safety and efficacy trials with excellent neutralizing capabilities against SARS-CoV-2, very rare reports of acute thrombotic thrombocytopenic events following administration emerged in certain populations, which triggered a series of clinical investigations that gave rise to a novel phenomenon called vaccine-induced immune thrombotic thrombocytopenia (VITT). Several converging pathways exist between VITT and other forms of thrombotic thrombocytopenic syndromes, specifically that of heparin-induced thrombocytopenia, which involves the formation of anti-PF4 antibodies and the activation of platelets leading to thrombocytopenia and thrombin-mediated clotting. Interestingly, certain differences in the presentation also exist in VITT, and guidelines have been published in recent months to assist clinicians in recognizing VITT to achieve desired outcomes. In this paper, we first discuss the clotting phenomenon in COVID-19 and delineate it from VITT, followed by a review of current knowledge on the clinical manifestations of VITT in lieu of other thrombotic thrombocytopenic syndromes. Likewise, emerging evidence on the role of adenoviral vectors and vaccine constituents is also discussed briefly.

## 1. Introduction

The ChAdOx1 nCoV-19 (Vaxzervia^®^) vaccine by AstraZeneca uses a simian-derived adenoviral vector encoding the codon-optimized full-length spike protein of SARS-CoV-2 [1]. Similarly, the Ad26.COV2.S vaccine (Janssen COVID-19) makes use of the Ad26 adenoviral vector to carry the spike protein gene and express it in cells bound by the non-replicating adenovirus [2]. Preliminary studies in millions of vaccinated individuals across various populations have shown significant vaccine efficacies for Vaxzervia^®^ (70.4%), in addition to its advantage of being cheaper and more accessible to developing countries, with an apparent increase in efficacy (90.0%) when given as a low-dose/standard-dose (LD/SD) two-dose regimen, according to pooled clinical trial results in the UK, South Africa, and Brazil [3,4]. This was shortly followed by the release of the Janssen COVID-19 vaccine by J&J, which was touted to be a more attractive and efficient option due to its one-dose regimen and excellent safety and efficacy profiles, preventing 66.1–66.9% of moderate or severe COVID-19 and up to 100% of hospitalizations compared with a placebo [5,6]. However, as surveillance reports in real-world studies would eventually reveal, very rare and unusual thrombotic thrombocytopenic complications in some populations taking the ChAdOx1 CoV-19 vaccine began to surface, stirring public health concerns. By March of 2021, many European countries issued decrees halting the administration of the said vaccine in favor of clinical investigation and research on vaccine efficacy against variants of concern and on reports of blood clotting events [7,8]. By April, several cases of cerebral venous sinus or splanchnic vein thromboses were reported by the European Medical Agency (EMA) through EudraVigilance, which led them to conclude that a very rare association between Vaxzevria^®^ vaccine exposure and the development of clotting disorders is present [9]. Subsequently, cases of thrombotic thrombocytopenia were also reported among patients vaccinated with Ad26.CoV2.S (Janssen), with anti-PF4 antibodies reportedly persistent for up to 5 months [9,10,11]. Current estimates show that the incidence of this novel and controversial phenomenon, later coined as vaccine-induced immune thrombotic thrombocytopenia (VITT), ranges from 1/26,500 to 1/127,300 persons and 1/518,181 persons vaccinated with the first and second doses of ChadOx1 nCoV-19, respectively. Meanwhile, the incidence of VITT is estimated to be at 1/263,000 persons vaccinated with a single dose of Ad26.COV2.S [12].

The phenomenon of VITT provides novel issues for patient care, vaccine development, and basic human physiology. Firstly, VITT remains a unique complication of adenoviral COVID-19 vaccines [13]. Secondly, the laboratory findings of VITT and spontaneous heparin-induced thrombocytopenia and thrombosis (HITT) are similar. They both show increased levels of anti-PF4 antibodies in the serum of patients with a thrombotic thrombocytopenic profile, warranting clarification and further investigation. Thirdly, VITT creates a new age risk group prone to developing the disease, which may lead to additional physical examination findings and management differences in patients suspected of having VITT.

The similarities between HITT and VITT lead to significant implications in patient disease and management and warrant a thorough investigation of their mechanisms for timely and effective treatment of the disease and its complications. However, little is known about VITT, and distinguishing it from the many syndromes of thrombotic thrombocytopenia can be difficult for inexperienced clinicians and researchers seeking to find treatment, despite the existence of recently published guidelines. In this paper, we therefore seek to demarcate the differences between VITT and other forms of thrombotic thrombocytopenia by first discussing the coagulation pathways in COVID-19 as a baseline comparison of disease. We then aim to differentiate the clinical manifestations and diagnosis of HITT from other related syndromes in relation to VITT. Lastly, we aim to briefly discuss the currently available evidence on the potential roles of adenoviral vectors, SARS-CoV-2 proteins, and other vaccine constituents in thrombosis, which is essential to determine possible targets for prevention or treatment.

## 2. The Clotting Phenomenon in COVID-19

Since the declaration of the COVID-19 pandemic in the year 2020, the volume and pace of SARS-CoV-2 research has been unprecedented. Recent studies have found that SARS-CoV-2 infection and subsequent COVID-19 are linked to the development of clotting disorders, which may be confused with the phenomenon underlying VITT in patients with both a history of adenoviral vaccine exposure and an active SARS-CoV-2 infection and must therefore be described. 

**Figure 1:**
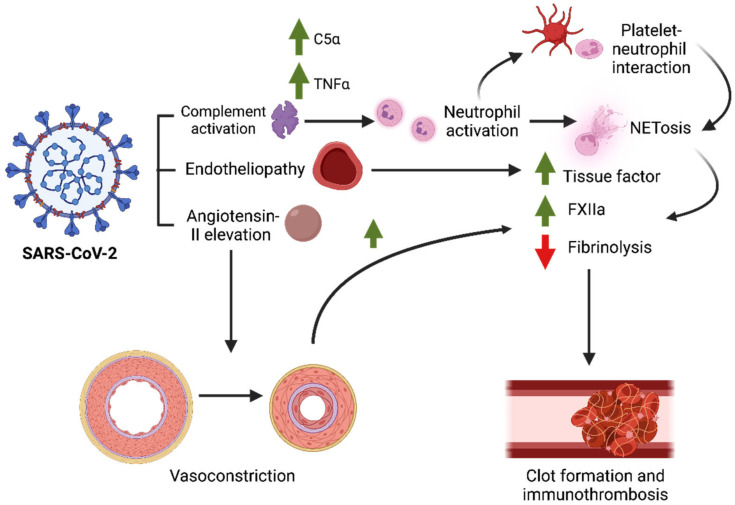
Mechanisms of SARS-CoV-2-induced thrombosis in severe COVID-19. The binding of spike protein to the ACE2 receptor site indirectly triggers several downstream events, including the activation of the complement pathway, endothelial injury, and vasoconstriction. Complement proteins activate neutrophils, which then release neutrophil extracellular traps (NETs) that act as a platform for contact activation of the coagulation cascade. Vasoconstriction secondary to elevated angiotensin-II promotes clot formation, which eventually lead to site-specific thrombosis in severe COVID-19.

As to how SARS-CoV-2 induces hypercoagulable, prothrombotic, and proinflammatory states is still under investigation. However, several preliminary mechanisms may already partly explain the thrombosis seen in COVID-19, which likely involve vascular and immune factors. During infection, SARS-CoV-2 binds to its human receptor, the angiotensin-converting enzyme-2 (ACE2) receptor, promoting viral entry inside the cell, which can lead to one of several events, including the downregulation of ACE2, increased levels of angiotensin II in plasma, or tissue injury [14,15]. As a response, chemokines such as MCP-1 and IL-8 are released, promoting the migration of immunologic and inflammatory cells to the damaged area. Neutrophils are recruited as first responders to inflammation at the injury site and are known to release neutrophil extracellular traps (NETs) in COVID-19 [16] (Figure 1). Biochemically, NETs are produced when neutrophils release their chromatin into the extracellular space as a form of cell death. They are usually made of intracellular components such as DNA, histones, highly cationic proteins, and some antimicrobial proteins (i.e., myeloperoxidase, neutrophil elastase) [17,18]. Most of these components are antigenic when released and lead to increased pro-inflammatory cytokines, including IL-1β, IL-6, IL-8, IFN-γ, and TNFα [18,19].

It is important to note that, although NETosis also occurs in other viral infections, such as those by the Chikungunya virus (CHIKV), respiratory syncytial virus (RSV), poxvirus, and human immunodeficiency virus (HIV) [20,21,22], it is primarily activated during a bacterial infection [23]. Likewise, it is unclear why neutrophils from SARS-CoV-2-infected individuals have a greater propensity for NETosis compared with those from non-infected individuals [24], which may be suggestive of neutrophil activation. A review by de Bont et al. suggests a possible mechanism for increased NETosis via crosstalks with the complement pathway. Briefly, microorganisms opsonized via C3b potently activate NETosis in neutrophils, with C5a and TNFα acting to recruit and prime neutrophils at the inflammation site. The activated neutrophils then produce complement factors C3, Factor B, and properdin, which activate the alternative complement pathway. The pro-inflammatory environment produced by complement activation combined with direct interactions between neutrophils and platelets leads to thrombus formation, highlighting the relationship between NETosis, thrombosis, and the complement pathway in some complications of COVID-19 [25,26,27,28]. One study suggests that NETosis may also promote contact activation of the coagulation cascade, which may explain the immunothrombosis phenomenon seen in severe COVID-19 cases [29]. Indeed, patients with severe COVID-19 usually have high neutrophil levels in lung tissue, as well as high neutrophil:lymphocyte ratios [30]. This is consistent with established knowledge on how NETosis can activate the coagulation cascade, which is via the production of tissue factor (TF), the activation of Factor XII, and the inhibition of fibrinolysis [31].

Aside from this immunologic response, the physical environment of the vasculature can also affect the clotting mechanism during SARS-CoV-2 infection. A study by Stalker et al. showed that the activities of clotting factors, including thrombin, are altered depending on the size of the wound and the vascular pressure in the injured area, which are important considerations during vascular injury due to feedback vasoconstriction [32]. Since angiotensin II levels in circulation directly affect vascular pressure by influencing the adrenal glands to produce aldosterone and via its breakdown products (Angiotensin 1 to 7) [15], it is also postulated that the renin–angiotensin–aldosterone system (RAAS) may play a pivotal role in COVID-19, which can possibly affect thrombosis based on the mechanisms discussed above (Figure 1). 

In COVID-19, there is documented evidence of a prothrombotic and proinflammatory state seen among severe and critically ill patients under hospitalization, marked by elevated levels of D-dimer and fibrinogen, prolonged prothrombin times, and the activation of proinflammatory cytokines, among others [33]. This leads to a hypercoagulable state that, when coupled with persistent inflammation, results in intravascular coagulation or thrombosis. Indeed, a study on 538 hospitalized COVID-19 patients in France found that the incidence of thrombotic complications in severe to critically ill COVID-19 patients was 22.7%, with 52% accounted for by pulmonary embolism [34]. Conversely, prophylactic anticoagulation reduced the risk of thrombosis without adversely affecting the risk of bleeding, confirming the presence of coagulopathy, and suggesting that this prothrombotic state responds to anticoagulation [34,35]. A study found that SARS-CoV-2 can infect endothelial cells, which may cause endotheliopathy, leading to vascular injury, and is made possible via the endothelial expression of ACE2 and TMPRSS2 [36,37]. This is consistent with studies providing histopathologic evidence of viral particles in renal endothelial cells and skin biopsies [38,39], as well as in vitro/vivo studies in mice showing endothelial infection by SARS-CoV-2 [40]. Another study suggests that SARS-CoV-2 utilizes paracellular transport from the epithelium to gain endothelial access and infect endothelial cells via interactions between the spike protein and the integrin αVβ3, which cause vascular dysregulation that is otherwise blocked by the integrin antagonist cilengitide [41]. However, these findings are disputed by contradicting studies showing that endothelial cells are either resistant to SARS-CoV-2 infection or are only infected when a high viral load is present [42,43]. Further, histological evidence suggesting absence of vascular endothelium infection by SARS-CoV-2 is also present in literature [44,45]. Hence, it is still unclear whether SARS-CoV-2 infection of the endothelium is a plausible mechanism for thrombosis in COVID-19, or whether thrombosis can be sufficiently caused by endotheliopathy alone or in combination with activation of the complement pathway, SARS-CoV-2 induced autoimmunity, or activation of the renin–angiotensin–aldosterone system [46]. 

To this end, how likely is it that COVID-19-induced thrombosis is contributory to VITT? There are similarities and differences between the thrombosis seen in COVID-19 and that observed in VITT. For instance, about 23% to more than half of thrombotic cases in COVID-19 are documented as pulmonary embolism, with other significant presentations being deep vein thrombosis (DVT), stroke, mesenteric or myocardial infarctions, among others [35,47,48]. Secondly, COVID-19 patients with pneumonia or severe disease are likely to have increased prothrombin times, which have also been consistently found to be higher among non-survivors [48]. Further, as aforementioned, D-dimer and fibrinogen levels are also elevated, with only a mild to moderate thrombocytopenia seen in severe COVID-19 infection, suggestive of coagulopathy and supported by findings of disseminated intravascular coagulation (DIC) in a large percentage (71–71.4%) of COVID-19 non-survivors [48,49,50]. Meanwhile, VITT presents with a different clinical presentation accompanied by different laboratory findings. Current reports show that VITT predominantly presents with neurologic signs as a result of venous thromboembolism in the central nervous system (CNS), as well as thrombosis in splanchnic or adrenal veins [51]. Likewise, VITT presents with moderate to severe thrombocytopenia while maintaining normal to only mildly elevated prothrombin times, elevated D-dimer levels, and hypofibrinogenemia [52].

While a retrospective study has shown that COVID-19 can also lead to a hundred-fold increased risk of developing CVST, thrombosis is commonly seen in severe and critically-ill COVID-19 cases, and studies comparing the risk of developing CVST between those with COVID-19 and those without COVID-19 but vaccinated with either ChAdOx1 nCoV-19 or Ad26.SARS2.S, to our knowledge, have not yet been performed [53,54]. In terms of treatment, anticoagulation with LMWH or unfractionated heparin (UFH) is preferred for thromboprophylaxis in COVID-19 patients who are at high risk for thromboembolism [55,56]. In contrast, management guidelines for VITT preclude the use of heparin, as is discussed below. Hence, COVID-19-induced thrombosis is clinically distinguishable from VITT and suggests that VITT is a separate clinical entity from the thrombotic complications of COVID-19. These clinical differences are summarized in Table 1. 

Meanwhile, a comprehensive review of the clinical presentation of VITT compared with other forms of thrombotic thrombocytopenia is discussed in the next section. Similarly, a summary of the mechanisms postulated to occur in VITT is summarized in Table 1 [57,58,59,60] but is discussed in detail in later sections.

## 3. Clinical Manifestations, Diagnosis, and Treatment of HITT and VITT in Relation to Other Thrombotic Thrombocytopenic Syndromes 

In many cases of thrombocytopenia, bleeding occurs due to a deficiency in circulating platelets, with clinical manifestations ranging from multiple petechiae or ecchymoses to spontaneous bleeding of the mucosa. In some cases, thrombocytopenia results from systemic factor activation that leads to a paradoxical increase in clot formation. This can be due to platelet activation by antiplatelet antibodies or by a defective von Willebrand factor (VWF), which result in aggregation and the inappropriate recruitment of other coagulation factors. A depletion of circulating platelets may then occur, which may explain paradoxical clotting in the setting of thrombocytopenia. Of the thrombocytopenic diseases known, paradoxical thrombosis occurs in heparin-induced thrombocytopenia (HIT), thrombotic thrombocytopenic purpura (TTP,) and hemolytic–uremic syndrome (HUS). We briefly discuss each syndrome in terms of their physiopathology, clinical manifestations, diagnoses, and treatments, followed by their similarities and differences with those of VITT. 

Heparin, or low-molecular-weight heparin (LMWH), which is normally given to patients requiring anticoagulation for prophylaxis or as a treatment for arterial or venous thromboembolism [61], acts by binding to the inhibitor antithrombin III to optimize its active site and increase its enzymatic activity [62]. In some cases, heparin binds to platelets and activates them, resulting in the release of platelet factor 4 (PF4) stored in its alpha granules, which are normally beneficial during vascular injury [63,64]. Unfortunately, PF4 can also be bound by heparin, and the PF4–heparin complex undergoes a conformational change to expose novel epitopes, which then lead to the formation of anti-PF4/heparin antibodies that are characteristic of HIT. The formed anti-PF4/heparin antibodies then bind the platelet’s FcγRIIa receptor, leading to further platelet activation [65,66], ultimately leading to platelet depletion and clotting, which are characteristics of heparin-induced thrombocytopenia with thrombosis (HITT).

Clinically, HIT does not lead to bleeding and thrombocytopenia is usually not severe [66], with deep vein thrombosis (DVT) being the most widely recognized manifestation, resulting in extremity gangrene, pulmonary embolism, or myocardial infarction in untreated cases [67,68]. This contrasts with the clinical manifestations of VITT, which include venous thrombosis of the CNS, adrenal, or splanchnic veins [51]. In addition, HIT is associated with exposure to low-molecular-weight heparin (LMWH) of unfractionated heparin (UFH), occurring within 5–14 days after exposure to heparin and is usually suspected using the 4T algorithm of (1) thrombocytopenia, (2) timing of decrease in platelet count, (3) thrombosis or localized skin reaction, and (4) non-evidence of other causes of thrombocytopenia. Although not all features may be present in a single patient, a score of 6–8 points suggest a high probability (about 50%) of diagnosing HIT, albeit with risks of overestimation in the ICU setting [66]. This phenomenon was formerly known as *Type II HIT,* where antibodies form against the PF4/heparin complex in autoimmune-mediated thrombotic thrombocytopenia. This was contrasted with *Type I HIT*, where heparin interacts directly with platelets, causing depletion via sequestration secondary to platelet clumping, which occurs within 48–72 h of heparin administration and does not involve an autoinflammatory reaction [69]. In contrast, a history of receiving an adenoviral vector-based vaccine for COVID-19 can prove to be essential information during history taking when considering a differential of HIT, such as VITT.

For confirmation, the presence of anti-PF4/heparin antibodies via ELISA or the ability of the patient serum to activate heparin–platelet solutions, called the platelet activation assay, typically supports the diagnosis with high specificity. In cases where a spontaneous HIT is suspected to occur, a platelet serotonin release assay (SRA) can be done in conjunction with or after performing an anti-PF4/heparin ELISA, which can support the diagnosis when peak serotonin release is greater than 80 percent at 0.1–0.3 U/mL heparin or greater than 50 percent in the absence of heparin exposure (0 IU/mL), in addition to at least two strongly positive PF4 enzyme immune assays (EIAs) and other characteristic features of HIT, such as inhibition at 100 IU/mL heparin or with Fc receptor-blocking antibodies in a patient who lacks a proximal history of heparin exposure [70]. 

For patients requiring anticoagulation for HIT, discontinuation of heparin and shifting to an alternative anticoagulant is warranted. To prevent thrombosis, a direct thrombin inhibitor (DTI) such as argatroban, an antithrombin pentasaccharide, such as fondaparinux, or the anti-Xa compound danaparoid may be used for antithrombosis, which are also given to VITT patients who require non-heparin anticoagulants. In the presence of thrombosis in HIT, patients can be treated with warfarin for 3 to 6 months, usually overlapped with a DTI or another antithrombin-binding compound such as fondaparinux to prevent venous gangrene [66]. In contrast, warfarin is contraindicated in VITT due to an increased risk of thrombosis or bleeding with no apparent benefit [71]. 

As opposed to HIT/T, thrombotic thrombocytopenic purpura (TTP) classically presents as a pentad in full-blown syndromes, characterized by microangiopathic hemolytic anemia, fever, thrombocytopenia, acute renal failure, and an altered mental status secondary to a seizure or stroke [72]. However, early detection in modern medicine has made it possible to no longer require the pentad in raising a high clinical suspicion for disease. 

Of the cases of TTP, the acquired form is more common and occurs more frequently in women, which is typically described by the presence of autoantibodies against the metalloprotease ADAMTS13, an enzyme that cleaves the von Willebrand factor (VWF). In contrast, acquired cases are described by a mutation or deficiency in the activity of ADAMTS13, which occur less frequently and are usually detected in childhood. Both forms of TTP accumulate uncleaved VWFs, which then promote platelet aggregation and thrombosis. Clinically, an increased lactate dehydrogenase (LDH), indirect bilirubin, and reticulocyte counts are seen, among others. In terms of treatment, plasma exchange for at least two days remains the mainstay of treatment for TTP to normalize the platelet count and resolve the signs of hemolysis [72]. Although plasma exchange can also be beneficial in HIT, the concept of platelet transfusion can prove fatal in this syndrome, as new platelets can provide substrates that can further aggravate thrombosis [73]. Meanwhile, plasma exchange therapy remains to be an effective option for some patients with VITT. 

A related, albeit less common, disease called hemolytic–uremic syndrome (HUS) can similarly present like TTP, with clinical findings such as acute renal failure (ARF), microangiopathic hemolytic fever, and thrombocytopenia preceded by diarrhea in typical cases caused by Shiga toxin-producing *Escherichia coli*. In atypical cases (aHUS), a mutation or autoantibody to factor H is more commonly seen, which prevents factor H-mediated inhibition of C3b and promotes the classical and alternative complement pathways in plasma-exposed cells [74]. As opposed to TTP and VITT, plasma exchange does not seem to affect the clinical outcomes of patients with HUS or aHUS, and treatment is primarily supportive, which may or may not include the anti-C5 antibody eculizumab [72]. 

As aforementioned earlier in this text, patients with VITT tend to present with neurologic signs (headache, visual disturbances, drowsiness), in addition to fever, mild bruising, and petechiae, as early as 4–28 days post-vaccination [67]. Similar to spontaneous HIT, antibodies against PF4 are produced without a proximal exposure to heparin, which current mechanisms suggest is a result of interactions between PF4 and vaccine constituents such as the adenoviral capsid and some non-assembled hexon proteins, promoted by pro-inflammatory molecules such as EDTA or possibly some trace human cell proteins [57]. Laboratory findings may include thrombocytopenia >15,000 cells/mm^3^ but which has been described to be as low as 7000 to 10,000 cells/mm^3^. In addition, elevated D-dimer levels by up to five times the upper normal limit are also seen, with a mild to moderate increase in variable international normalized ratios (INRs). More importantly, the presence of anti-PF4 antibodies as early as 7 to 10 days post-COVID-19 vaccination, to as late as 24 to 30 days in delayed presentations, is usually documented [73,75]. Although laboratory findings in VITT resemble that of disseminated intravascular coagulation (DIC), such as normal to decreased fibrinogen, elevated D-dimer levels, and moderate to severe thrombocytopenia, contrasting features include a predominance of thrombosis in VITT and a normal to mildly elevated PT/aPTT or INR [51,72]. To date, guidelines established by credible medical associations to formalize the diagnosis and management of VITT include those published by the National Institute for Health and Care Excellence (NICE) and the American Society of Hematology, which are summarized in Table 2 [76,77].

In terms of treatment, current management algorithms for VITT are similar to HIT: administration of a non-heparin anticoagulant to address thrombosis (argatroban, fondaparinux, danaparoid), plasma exchange therapy to deplete the plasma of the pathologic anti-PF4 antibodies and to address the thrombocytopenia and hypofibrinogenemia, use of glucocorticoids, and IV immunoglobulin G (IVIG) to compete with the anti-PF4 antibodies to block platelet activation, which is usually given to patients with autoimmune or heparin-independent HIT at a dose of 1 g/kg for at least two days [73,78]. Similarly, anecdotal reports of managing patients with VITT have shown that administration of LMWH (0.1–0.3 U/mL up to 100 U/mL) combined with endovascular recanalization of the venous sinuses in CVST can normalize platelet counts and improve patient survivability [74,79]. However, due to the lack of robust and high-quality evidence on the benefit of LMWH, interim recommendations suggest against heparin administration in patients with suspected or confirmed VITT [80]. It is important to note that treatment can be started prior to confirmation of ELISA and SRA results if there is a high clinical suspicion of VITT (severe symptoms, thrombocytopenia, positive imaging, elevated D-dimer levels five times the upper normal limit) [81]. For more information on the approach to treating patients with VITT, the reader is referred to current guidelines and recommendations [71,81]. 

A summary of the clinical manifestations, diagnosis, and treatment of HITT and VITT to other thrombotic thrombocytopenic syndromes is listed below (Table 2) [76,77]. Briefly, VITT is diagnosed when the following five criteria are met: (1) COVID-19 vaccination 4 to 42 days (5 to 30 days for NICE) prior to symptom onset, (2) venous or arterial thrombosis (cerebral or abdominal) via same-day CT imaging, (3) thrombocytopenia with platelet counts < 150 × 10^9^/L, (4) positive anti-PF4 ELISA, and (5) a markedly elevated D-dimer greater than four times the upper limit of normal (>4000 µg/L FEU or DDU for NICE) [76,77]. These criteria are also highlighted in Table 2.

## 4. The Role of Adenoviral Vectors and Vaccine Constituents in VITT

The previous section discussed the distinguishing features of VITT and thrombotic thrombocytopenic syndromes with similar thrombotic thrombocytopenic syndromes. An interesting finding among patients diagnosed with VITT is the unique exposure to the two adenoviral vector-based COVID-19 vaccines, ChAdOx1 nCoV-19 (Vaxzervia^®^) and Ad26.COV2.S (Janssen), about 7–10 days but up to 30–42 days post-vaccination. This association, documented and announced by the EMA last April of 2021, is unique only to the adenoviral vector vaccines for COVID-19, which are otherwise not found to occur in other COVID-19 vaccine types (mRNA, nanoparticle, or inactivated vaccines). More importantly, the other adenoviral vector-based vaccines manufactured to date (the first dose of the rVSV-ZEBOV Ebola vaccine) and those in present clinical trials (HIV, tuberculosis, malaria adenoviral vaccines) have not reported thrombotic thrombocytopenia as a rare complication of administration. Although there is no established evidence on the physiopathology of VITT, present associations between exposure to the adenovirus and the development of thrombosis and studies on the role of adenoviral vectors in thrombosis have indeed been published in the literature.

A study by Zhang et al. sought to determine whether human platelets directly interacted with the SARS-CoV-2 spike protein. Using platelets from healthy volunteers and COVID-19 patients, they showed that human platelets expressed hACE2 and TMPRSS2, and platelets from COVID-19 patients had notable hyperactivity and mean platelet volumes correlating with decreased overall platelet counts [82]. These suggest that SARS-CoV-2 or its spike protein can directly interact with platelets and activate them, possibly being the primary mechanism for thrombotic complications in moderate to severe COVID-19 or in VITT. However, this study deviates from the current understanding that platelets do not express ACE2, and additional studies may be needed to establish this expression profile. 

Another study by Baker and colleagues used computational simulations to show that the viral capsid of ChAdOx1 binds with PF4 through the spaces of its hexon proteins, potentially promoting the formation of anti-PF4 antibodies and partially explaining the presence of platelet-activating anti-PF4 antibodies in ChAdOx1 nCoV-19-vaccinated individuals [83,84]. In addition, several articles have shown that adenoviruses can by themselves bind to platelets in vitro or shortly after administration in mice, resulting in platelet aggregation and thrombocytopenia [83,84,85]. A more recent study combining in vitro and in vivo approaches in mice also found that the components of the ChAdOx1 nCoV-19 vaccine (including the hexon protein of the adenovirus) formed complexes with PF4, and injection of the vaccine caused vascular leakage in mice, which may be important mechanisms for platelet and neutrophil activation in VITT [58]. Other studies suggest that EDTA and trace human cell line proteins present in these adenoviral COVID-19 vaccines also induce a pro-inflammatory state that can contribute to VITT; however, it is still unclear whether these directly contribute to pathogenesis [57]. To our knowledge, many of the studies so far do not provide strong evidence of direct adenoviral involvement or involvement of non-viral constituents, and stronger evidence for the mechanism beyond preliminary studies is needed. On a similar note, the predilection of younger age groups to develop VITT as a rare complication of vaccination remains to be investigated and warrants further research on risk factors, including genetic predisposition, hormonal influences, and factors related to differences in ethnicity.

## 5. Conclusions

Understanding the basic mechanisms underlying coagulation pathways is essential for managing novel thrombotic complications. The newly recognized vaccine-induced immune thrombotic thrombocytopenia (VITT) secondary to immunization with adenoviral vector-based COVID-19 vaccines has given rise to interesting questions on the role of adenoviral vectors and coronavirus proteins in thrombosis and how they can contribute to the development of VITT. Current evidence shows that VITT is a very rare complication of COVID-19 vaccination with ChadoX1 CoV-19 and Ad26.COV2.S, with estimates of incidence ranging from 1/26,500 persons to 1/518,181 persons vaccinated with ChadOx1 nCoV-19 and 1/263,000 persons vaccinated with Ad26.COV2.S. VITT has a predilection for venous thrombosis in the CNS, splanchnic or adrenal veins, with patients presenting neurologic signs in addition to fever and mild bruising as early as 4–28 and up to 30 days post-COVID-19 vaccination. Differential diagnoses commonly include HIT and, less commonly, microangiopathies such as TTP and HUS. Treatment for VITT requires non-heparin anticoagulants as in HIT, with options such as IVIG for blockage of anti-PF4 antibodies and plasma exchange therapy as in TTP. Future investigations should focus on determining alternatives for thrombosis-promoting constituents to improve the efficacy and safety profiles of future COVID-19 vaccines.

## Figures and Tables

**Table 1:** Brief comparison between COVID-19 thrombosis and VITT.

	COVID-19 Thrombosis	VITT
Pathophysiology	- Angiotensin II elevation - Endotheliopathy - Complement activation - NETosis and platelet activation	- Working mechanism: Adenoviral capsid-PF4 or hexon-PF4 interactions - Vaccine trace constituents (EDTA, trace cell proteins) possibly promoting inflammation but requires more evidence - Non-viral binding partners still unclear - Formation of anti-PF4 antibodies
Hemostatic abnormalities	- Seen in severe COVID-19 usually 6 to 21 days after ICU admission - Elevated prothrombin time, D-dimer, and fibrinogen - Mild thrombocytopenia	- Seen 4–28 days after adenoviral COVID-19 vaccination - Normal to mildly elevated prothrombin time - Elevated D-dimer - Decreased fibrinogen - Moderate to severe thrombocytopenia
Complications	- Pulmonary embolism, VTE, DVT, stroke, mesenteric or myocardial infarction - Uncommon CVST	- CVST, thrombosis of adrenal or splanchnic veins - Uncommon DVT, stroke, or MI
Treatment	- Parenteral LMWH (preferred) or unfractionated heparin (UFH) - No benefit in oral anticoagulants for thromboprophylaxis (NIH)	- Non-heparin anticoagulants (argatroban, fondaparinux, or danaparoid), - IVIG, plasma exchange therapy - Case-to-case glucocorticoid use

**Table 2:** Clinical features, diagnosis, and treatment of thrombotic thrombocytopenic syndromes.

	VITT	HITT	TTP	HUS/aHUS
Mechanism	- Adenoviral capsid interacts with PF4 - Non-assembled hexon proteins also interact with PF4 - Non-viral binding partners currently unclear - Leads to formation of anti-PF4 antibodies	- LMWH binds to platelets causing the release of PF4 - LMWH also binds to PF4, leading to anti-PF4/heparin antibody production - Leads to platelet aggregation and thrombosis - In heparin-independent HIT, anti-PF4 antibodies are formed	- Acquired: antibodies more commonly form against ADAMTS13 - Hereditary: mutation or deficiency in ADAMTS13 less common - Retains uncleaved Von Willebrand Factor (VWF) - Both promotes platelet aggregation and thrombosis	- Typical HUS: Shiga toxin-producing *E. coli* causes an immune response to factor H, promoting activation of C3b - Atypical HUS: mutations or autoantibodies to key factors (H, I) prevent C3b inhibition
Clinical manifestations	- Neurologic signs (headache, visual disturbances, drowsiness) - Bruising or petechiae with fever; less likely are signs and symptoms of DVT - Occurs 7–10 days after vaccination with ChAdOx1 nCoV-19 (AstraZeneca) or Ad26.COV2.S (Janssen) COVID-19 vaccines - Delayed detection usually up to 24 to 30 days post-vaccination	- 4T algorithm: thrombocytopenia without bleeding, timing within 5–14 days after heparin administration, thrombotic signs and symptoms (significant Wells score or signs of DVT, skin necrosis at heparin injection site), other causes of thrombocytopenia excluded	- Characteristic pentad: jaundice or pale conjunctiva (microangiopathic hemolytic anemia), purpura or ecchymoses (thrombocytopenia), renal failure, neurologic findings (headache, dizziness, seizures, visual disturbances), fever - May develop some but not all clinical signs; pentad not required for diagnosis	- Microangiopathic hemolytic anemia - Thrombosis resulting in acute renal failure, seizure, or stroke - Signs of uremia, bruising, petechiae, or ecchymoses (thrombocytopenia) - Fever, usually preceded by hemorrhagic diarrhea in typical HUS - Can be difficult to distinguish from TTP in atypical cases
Diagnosis	- COVID-19 vaccination 5–30 days (NICE) or 4–42 days (ASH) prior to symptom onset - Any arterial or venous thrombosis - Thrombocytopenia (<150 × 10^9^/L) - Presence of anti-PF4 antibodies via ELISA - Elevated D-dimer (>2000–4000 µg/L FEU/DDU) and normal to decreased fibrinogen (>2–4 g/L) - Additional features: normal to mildly elevated PT/aPTT and INR - PF4 antibody testing (confirmatory assay)	- HIT/HITT is a clinical diagnosis. Supporting lab findings include mild to moderate thrombocytopenia (<20,000/mm^3^), normal PT/aPTT and INR, anti-PF4/heparin antibodies via ELISA, positive platelet activation (serotonin release) assay, 5–14 days after exposure to heparin in classical HIT - Thrombotic thrombocytopenia without heparin exposure, strong SRA > 50% at 0IU/mL heparin, strong positivity in at least two PF4 enzyme immune assays in spontaneous HIT	- Thrombocytopenia - Normal PT/aPTT, INR - Increased LDH, indirect bilirubin, and reticulocyte counts - Decreased hematocrit - Deficiency in ADAMTS13 activity (<10%) and presence of anti-ADAMTS13 antibodies	- Thrombocytopenia, - Normal PT/aPTT, INR - Normal to decreased ADAMTS13 activity - Reduced eGFR - BUN: CREA > 20 or azotemia with or without uremic signs and symptoms
Treatment	- Non-heparin anticoagulants (argatroban, fondaparinux, danaparoid) - IVIG - Case-to-case use of glucocorticoids - Plasma exchange therapy	- Discontinuation of heparin or shift to non-heparin anticoagulation (argatroban, danaparoid) - Warfarin overlapped with direct thrombin inhibitors after resolution of thrombocytopenia	- Plasma exchange therapy, rituximab, or caplacizumab - Immunomodulatory therapies: cyclophosphamide, vincristine - Splenectomy	- Supportive only; plasma exchange therapy does not affect clinical outcomes - Eculizumab in patients vaccinated against meningococcemia
Complications	- Venous thrombosis in the central venous sinus (most common) - Thrombosis of splanchnic or adrenal veins - DVT (less common) - Arterial thrombosis also occurs and uncommonly leads to stroke or MI	- Commonly progresses to HITT if left untreated, manifesting as deep vein thrombosis with possible pulmonary embolism or MI - Extremity ischemia or gangrene may lead to amputation	- Thrombotic microangiopathy results in renal failure, retinopathies, seizures, or stroke - Petechiae or ecchymoses in the skin are also seen	- Thrombotic microangiopathy results in renal failure, retinopathies, seizures, or stroke - Petechiae or ecchymoses in the skin are also seen

## Abbreviations

AZ	AstraZeneca
ChAdOx1	Chimpanzee (Ch) Adenovirus-vectored vaccine (Ad) University of Oxford (Ox) 1
COVID-19	Coronavirus Disease 2019
CVST	Central Venous Sinus Thrombosis
HIT	Heparin-Induced Thrombocytopenia
HITT	Heparin-Induced Thrombocytopenia and Thrombosis
HUS	Hemolytic Uretic Syndrome
J&J	Johnson & Johnson
LMWH	Low-Molecular-Weight Heparin
SARS-CoV-2	Severe Acute Respiratory Syndrome Coronavirus 2
TTP	Thrombotic Thrombocytopenic Purpura
UFH	Unfractionated Heparin
VITT	Vaccine-Induced Immune Thrombotic Thrombocytopenia
VWF	Von Willebrand Factor
